# Study on the Compromise Effect Under the Influence of Normative Reference Group

**DOI:** 10.3389/fpsyg.2022.869551

**Published:** 2022-04-15

**Authors:** Qinglong Du, Kunyang Li

**Affiliations:** School of Economics and Management, Southwest University of Science and Technology, Mianyang, China

**Keywords:** reference group, compromise effect, consumer choice, experimental study, group norms

## Abstract

The compromise effect is an important context effect, but its research is still insufficient under the influence of social factors and purchase tasks. This study explores the change of compromise effect in different group norm scenarios by constructing three different group norm reference points. Three conclusions were drawn. First, the compromise effect always exists under the influence of different groups’ normative reference points if there is a compromise effect in a product set. Second, the effect value of the compromise effect will be significantly different with the change of group norm reference point. Third, group norms can indeed induce the compromise effect. Therefore, these findings would help to further enrich the literature results of the compromise effect and strengthen its application in marketing practice.

## Introduction

Consumers often face a choice set composed of multiple products when making purchase decisions. The probability of a product being selected often depends on the relative position of the product in a specific selection set (Novemsky et al., 2007), resulting in some context effect (or Decoy effect), such as compromise effect, attraction effect, and perceptual focus effect (Simonson, 1989; Simonson and Tversky, 1992; Reb et al., 2018; Banerjee et al., 2020). The compromise effect is one of the important context effects. It poses a severe challenge to the traditional preference consistency assumption and has important theoretical significance. At the same time, it is also widely used in product display, advertising, and other marketing practices. Although previous studies have carried out a lot of research on the occurrence mechanism and influencing factors of the compromise effect, relevant studies mainly focused on individual factors (Simonson, 1989), product factors (Mourali et al., 2007; Yoo et al., 2018), and purchase process (Hamilton et al., 2007), ignoring the social environment, purchase tasks, and etiquette situations. In addition, during the research process, some scholars believe that the experimental conditions of context effect are very difficult to achieve, so its effectiveness in practice is questionable (Frederick et al., 2014; Yang and Lynn, 2014).

Huber et al. (1982) considered that “group norms” may be one of the reasons for the context effect when studying the attraction effect. Simonson (1989) also incorporated the influence factors of the reference group in the study of the compromise effect. For example, in the experiment, subjects were asked to consider the evaluation of their classmates on the results of their choice behavior, but some of his hypotheses were not fully confirmed. According to the reference group theory, the product selection is more vulnerable to the norms of the reference group only when the product has the characteristics of public or luxury (Bearden and Etzel, 1982). By analyzing the experimental process of Simonson (1989), it can be found that the products selected in the experiment do not meet the characteristics of display or luxury, which may be an important reason why his hypothesis has not been fully verified. Therefore, the choice of experimental products is very important because it may affect the experimental results. This study measures group norms based on the perspective of face in the Chinese context, because consumers will be affected by the norms of the reference group when they are involved in “face” consumption decisions, to determine the appropriate test scenario. In conclusion, further research on the compromise effect under the influence of reference group norms will help to further enrich the literature results of the compromise effect and strengthen its application in marketing practice.

## Literature Review and Hypothesis

### Compromise Effect

Simonson (1989) put forward the concept of compromise effect based on the research of Huber et al. (1982). When a product becomes a compromise product or intermediate product, even if there is no advantage relationship, its possibility of selection will rise. Specifically, if the third product C is added to the core product sets A and B, compared with product B, product C is not at a disadvantage, but the market share of product B has increased significantly. This phenomenon is the so-called compromise effect. That is, the compromise effect occurs when products change from extreme products to intermediate products (Khan et al., 2011).

The compromise effect originates from the changing process of consumers from standard conflict to standard reconciliation in the selection process, so its occurrence is a complex cognitive process (Dhar and Simonson, 2003). Some factors may lead to the phenomenon of compromise choice, such as complexity of decision-making (Simonson, 1989; Novemsky et al., 2007), consumer choice motivation (Mourali et al., 2007), and product attribute imbalance (Simonson and Tversky, 1992). The method proposed by Mourali et al. (2007) can be used to calculate the compromise effect value. If A is a competitive product, B is a target product, and C is a temptation product, P(A;B,C) and P(B;A,C) represent the selected share of products A and B in the extended set {A,B,C}, respectively, and P_*C*_ (B; A) represents the selected share of product B relative to product A in the extended set {A,B,C}, then:


$$P_{\text{C}}\,\left(B;A\right)=\frac{P\,\left(B;A,C\right)}{P\,\left(B;A,C\right)+P\,\left(A;B,C\right)}$$

Then the compromise effect value (generally calculated by the difference of proportions) is equal to:


$$\Delta \,P_{\text{B}}=P_{\text{C}}\,\left(B;A\right)-P\,\left(B;A\right)$$

Whether ΔP_B_ is significant can be tested by the chi-square test. Since the compromise effect obeys binomial distribution, according to the large sample normality principle of maximum likelihood estimation parameters, Z statistics can also be constructed for testing.

### Reference Group and Its Normative Influence

Simonson (1989) believes that when consumers face decision-making difficulties and “correct” their behavior, they will increase their preference for compromise products. Mourali et al. (2007) objected to this. They believed that the “correction” of consumers’ motivation orientation was the key reason for the formation of the compromise effect. Normative influence may lead to group punishment for consumer’s choice (Childers and Rao, 1992). Loss aversion may change consumer’s choice motivation orientation, such as forcing them to shift from pursuing product attribute function to pursuing product social function (White and Dahl, 2006). To please the reference group, consumers buy more “safe” compromise products (Simonson, 1989) to avoid being criticized by the reference group. For Chinese people who prefer the golden mean (middle is the right choice in the world; normal is the fixed word on earth), making a compromise choice may itself be a potential social norm.

It is assumed that the following product preference order “*A*≺*B*≺*C*” meets the group specification. When the product specifications (such as quality and price) selected by consumers are not lower than the specifications of the reference group, we call it “specification consistency” behavior. Suppose there is a product set {A, B, C} with a compromise effect, where A is a competitive product, B is a target product, and C is a temptation product. The cost that consumers need to pay for purchasing the product (including price, time, and physical strength) is A < B < C. When consumers make a consumption, the preference order of referring to the group norms is *A*≺*B*≺*C*. We establish the following analysis framework.

### Reference Group Norms Take Competitive Products as the Reference Point

According to the reference dependence theory (Tversky and Kahneman, 1991), the consumer’s choice of any product in the product set is a “normative consistency” behavior when the group norm takes the competitive product (product A) as the reference point. When consumers choose product A, the benefits obtained by them include the benefits of product function. When choosing product B or C, consumers will not only get the functional benefits of the product but also will pay higher purchase costs. Because the purchase of product A is in line with the norms, according to the principles of loss aversion and diminishing sensitivity (Tversky and Kahneman, 1992), whether consumers buy product B or C will not be constrained by the group norms. That is, in the scenario where the group norms are very easy, consumers will mainly choose according to the product attributes rather than the group norms. Therefore, we propose the following hypotheses.

Hypothesis 1: The compromise effect still exists when the reference group norms take product A (competitive product) as the reference point.
Hypothesis 2: The compromise effect value will not change significantly when the reference group norms take product A (competitive product) as the reference point.

### Group Norms Take Core Products as the Reference Point

When the group norm takes product B as the reference point (that is, choosing product B or C is the “norm consistency” behavior), if consumers choose product A, their group image income with “face” as the core is negative (White and Dahl, 2006). If consumers are sensitive to the normative impact of the group and based on loss aversion, they will give up the choice of product A. Choosing product B is in line with the norms and the “golden mean” of Chinese people in terms of group norms. Therefore, consumers will not cater to product C which is more expensive. Under the joint action of compromise effect and group norms, the market share of competitive product (product A) will be lost, and the compromise effect will be significantly strengthened. Therefore, we propose the following hypotheses.

Hypothesis 3: The reference group norms will strengthen the compromise effect and the compromise effect will increase significantly when the group norms take the target product (product B) as the reference point.
Hypothesis 4: The effect value of the compromise effect reaches the maximum when the group norms take the target product (product B) as the reference point.

### Group Norms Take Temptation Products as the Reference Point

When the group specification takes product C (temptation product) as the reference point, because a certain attribute of product C is obviously different from products A and B, whether consumers choose product A or B is not the behavior of “specification consistency.” Due to loss aversion, group norms will significantly reduce the probability of selecting the target product (product B) and another endpoint product (product A), and significantly increase the probability of selecting the temptation product (product C), resulting in another extreme reversal–polarization phenomenon (Simonson and Tversky, 1992). Since the competitive product (product A) deviates furthest from the group norms, the market share of product A suffers more than that of product B, which will lead to the existence of the compromise effect. Therefore, we assume that:

Hypothesis 5: The compromise effect still exists when the reference group norms take the temptation product (product C) as the reference point.
Hypothesis 6: The group norms pressure will lead to the polarization of preference for temptation products in the selection results when the reference group norms take the temptation product (product C) as the reference point.

## Methods and Results

### Pilot Study

The pre-test mainly tests whether the product selection can distinguish different group norms pressure. Through discussion with college students (majoring in Marketing), they believe that restaurant hospitality can better show the public and luxury characteristics of Bearden and Etzel (1982). Through the research conclusion of Li and Su (2007) on the relationship between “face” and reference group norms, students believe that they can choose the “dining environment” and “total consumption amount” of restaurants to test the pressure of reference group norms faced by consumers when choosing restaurants. Referring to the compromise effect experiment of Simonson (1989), the students recommended three restaurants around the school (restaurants A, B, and C). We approximate the dining environment of three restaurants provided by students (0 = worst and 100 = best) and the total consumption amount of two people (unit: RMB) to form the following ternary product set (Table 1).

**TABLE 1:** Experimental product set and product attribute composition.

Product set	Product attribute	
	
	Dining environment (0 = worst 100 = best)	Total consumption amount
Restaurant A	70	100
Restaurant B	80	180
Restaurant C	90	260

Then, we invited 123 college students to conduct a questionnaire test on the group norms pressure of restaurant selection. The questionnaire contains three test scenarios: “(scenario 1) suppose that classmate Zhang chose restaurant B for dinner and classmate Li chose restaurant A for dinner. Please choose classmate Li’s treat behavior according to your own feelings and in combination with the Chinese view of the face: 1 = lose face; 2 = fight for face; and 3 = up to face.” Scenario 2 and scenario 3 replace restaurant A in scenario 1 with restaurant B and restaurant C, respectively. The experimental design adopts the between-group experiment, and the test results are shown in Table 2. When classmate Zhang treats to restaurant B, if classmate Li chooses restaurant A, 82.93% of the subjects think it is a kind of “losing face” (lower than the group norm). If choosing restaurant B, 85.37% of the subjects thought it was a kind of “fight for face (in line with group norm).” If choosing restaurant C, 80.49% of the subjects think it is an “up to face” behavior (higher than the group norm).” Therefore, the product set in Table 1 can be used to represent three different group consumption norms (χ^2^ = 417.64 and *p* < 0.01).

**TABLE 2:** Consumer’s perception of group norm pressure when the reference point is product B.

Group norm pressure		Restaurant A	Restaurant B	Restaurant C
Lower than the group norm	Losing face	102 (82.93%)	6 (4.88%)	0 (0.00%)
In line with group norm	Fight for face	18 (14.63%)	105 (85.37)	24 (19.51%)
Higher than the group norm	Up to face	3 (2.44%)	12 (9.76%)	99 (80.49%)
Total		123 (100%)	123 (100%)	123 (100%)

### Study 1

#### Method

This study mainly tests whether there is a compromise effect in the experimental product set. To meet the requirements of the reference group’s norms on products (or services), we chose the restaurant in the pre-experiment as the experimental product. Referring to the experimental process of Simonson (1989) and Chuang and Yen (2007), we verify the compromise effect and set up a binary product set {restaurant A, restaurant B}, including competitive products and target products. On this basis, temptation products are added to form a ternary product set {restaurant A, restaurant B, restaurant C}. The subjects were shown two attributes of the restaurant: the dining environment and the total amount of consumption for two people (Table 3).

**TABLE 3:** Compromise effect experimental product set.

Restaurant name	Binary product set		Ternary product set	
		
	Dining environment	Price (RMB)	Dining environment	Total consumption amount (RMB)
Restaurant A	70	100	70	100
Restaurant B	80	180	80	180
Restaurant C			90	260

#### Manipulation

We set up two experimental scenarios. Scenario 1 is: “if you invite a classmate to dinner, there are two restaurants A and B to choose from. Except for the difference between the dining environment (0 = worst and 100 = best) and the total amount of consumption, other aspects are similar (such as dish quality and hygiene conditions), which restaurant would you choose?” Scenario 2 expands the restaurant into three restaurants A, B, and C. To avoid the significant influence of the “correction” behavior of the subjects on the compromise effect (Simonson, 1989), a between-group design was used in the experiment. A total of 60 volunteers were recruited and randomly assigned to two experimental scenarios. Subjects choose products according to their experimental scenario.

#### Results

The product selection results of consumers under scenario 1 (binary product set) and scenario 2 (ternary product set) are shown in Table 4. In scenario 1, the probability of subjects choosing restaurant B is p (B; A) = 20%. After adding temptation product restaurant C (scenario 2), the probability of subjects choosing restaurant B is p (B; A, C) = 53.3%, and the probability of choosing restaurant A is p (A; B, C) = 36.7%. Then in the extended ternary product set {A, B, C}, the selected share of product B relative to product A is:


$$P_{\text{C}}\,\left(B;A\right)=\frac{P\,\left(B;A,C\right)}{P\,\left(B;A,C\right)+P\,\left(A;B,C\right)}=\frac{53.3\%}{53.3\%+36.7\%}=59.2\%$$

**TABLE 4:** Consumer choice statistics of target products.

Restaurant name	Dining environment	Price (RMB)	Dining environment	Total consumption amount (RMB)
Restaurant A	70	100	24 (80.0)	11 (36.7)
Restaurant B	80	180	6 (20.0)	16 (53.3)
Restaurant C	90	260	—	3 (10.0)
Total	—	—	30 (100)	30 (100)

*The data in brackets are percentages (%).*

Compromise effect = P_*C*_ (B; A)–P (B; A) = 39.2% (χ^2^(1) = 9.24, *p* = 0.002), that is, there is a compromise effect in the extended product set {A, B, C}.

### Study 2

#### Method

This study mainly tests the influence of reference group norms on the compromise effect. According to the purpose of the study, three experimental groups were set up, taking restaurants A, B, and C as the reference point of group norms. Since the reference group norms are easily regulated by consumers’ normative susceptibility and attention to social comparative information, we first tested the “consumers’ susceptibility (norms)” and “attention to social comparative information” of the sample, in which consumers’ susceptibility is measured by Schroeder (1996). Paying attention to social contrast information was tested with the scale of Bearden et al. (1990). According to the test results, the samples were randomly assigned to three experimental groups.

#### Manipulation

Three experimental scenarios were simulated, and three restaurants were used as the reference point of group norms. Scenario 1 takes restaurant A as the reference point of group norms: “if you invite a classmate (or friend) to dinner, there are three restaurants A, B, and C to choose from. Except for the difference in dining environment (the highest score is 100 points) and total consumption amount, other aspects are similar (such as dish quality and sanitary conditions). If the classmate (or friend) usually goes to a restaurant with similar conditions as A, which restaurant would you choose?” Scenario 2 (the reference point of group norms is restaurant B) and experiment scenario 3 (the reference point of group norms is restaurant C) are similar to experiment scenario 1, except that the norm is set to “if the student (or friend) goes to a restaurant with similar conditions as B (or C), which restaurant would you choose for dinner?” A total of 106 undergraduates (all samples did not participate in the prediction test and study 1) were recruited to participate in the experiment.

#### Results

When the group norm takes restaurant A as the reference point, the subject’s choice of restaurants is shown in Table 5. The probability of restaurant B being selected is p (B; A, C) = 63.3%. In the extended set {A, B, C}, the selected share of restaurant B relative to restaurant A is P_*C*_ (B; A) = 63.3%, and the compromise effect = P_*C*_ (B; A)–P (B; A) = 43.3% (χ^2^(1) = 11.58, *p* < 0.01). That is, when the group norm takes restaurant A as the reference point, the compromise effect is still significant. Therefore, hypothesis 1 was supported. The change value of compromise effect = 43.3–39.2% = 4.1% (χ^2^(1) = 0.10, *p* < 0.75), that is, the change of effect value is not significant. Therefore, hypothesis 2 was supported.

**TABLE 5:** Consumer choice statistics of target products.

Restaurant name	Dining environment	Price (RMB)	Group norm reference point		
			
			Reference point 1	Reference point 2	Reference point 3
Restaurant A	70	100	11 (36.7)	1 (3.23)	5 (14.28)
Restaurant B	80	180	19 (63.3)	26 (83.87)	11 (31.43)
Restaurant C	90	260	0 (0.0)	4 (12.90)	19 (54.29)
Total	—	—	30 (100)	31 (100)	35 (100)

*The data in brackets are percentages (%).*

When the group norm takes restaurant B as the reference point, the probability of restaurant B being selected is p (B; A, C) = 83.9%. In the extended set {A, B, C}, the selected share of restaurant B relative to restaurant A is P_*C*_ (B;A) = 96.3%, and the compromise effect = P_*C*_ (B; A)–P (B; A) = 76.3% (χ^2^(1) = 33.60, *p* < 0.01). That is, when the group norm takes restaurant B as the reference point, the compromise effect is significant. The change value of compromise effect = 76.3–39.2% = 37.1% (χ^2^(1) = 10.71, *p* < 0.01), that is, the effect value has changed significantly. Therefore, hypothesis 3 was supported.

When the group specification takes restaurant C as the reference point, the probability of restaurant B being selected is p (B; A, C) = 31.4%, the share of product B relative to product A in the extended set {A, B, C} is P_*C*_ (B; A) = 68.75%, and the compromise effect = P_*C*_ (B; A)–P (B; A) = 48.75% (χ^2^(1) = 10.64, *p* < 0.01), that is, the compromise effect still exists. Therefore, hypothesis 5 was supported.

In addition, D_*C*_(B) = P_*C*_(B;A)-P(B;A) = 68.75–20% = 48.75% > 0, D_*B*_(A) = P_*B*_(A;C)-P(A;C) = 20.8–83.3% = −62.5% < 0, D_*A*_(B) = P_*A*_(B;C)-P(B;C) = 36.7–66.7% = −30.0% < 0. For example, D_*C*_(B) measures the degree to which the addition of C to the set {B, A} changes the relative popularity of B and A. Based on this, if the group norm takes restaurant C as the reference point, the subject’s choice behavior shows the polarization phenomenon of preference for restaurant C. Therefore, hypothesis 6 was supported. This shows that when the group norms prefer luxury goods, consumers have a very strong desire to consume luxury goods to improve their face (Li and Su, 2007), rather than making choices based on product attributes.

The above results show that when the group norm takes the target product restaurant B as the reference point, the compromise effect reaches the maximum (76.3%), which is significantly higher than that of the competitive product restaurant A (Δ*P*1 = 33.0%, *t* = 2.79, and *p* < 0.01) and temptation product restaurant C (Δ*P*2 = 27.6%, *t* = 2.42, and *p* < 0.01) is the value of compromise effect at the reference point. Therefore, hypothesis 4 was supported, which shows that the compromise effect can be induced by social factors, such as group norms.

## Discussion

The conclusion shows that for a group of product sets with compromise effect, even under the influence of social factors (such as reference group norms), the target products are still easier to choose than competitive products, which shows that the compromise effect has strong stability (Simonson, 1989). However, the effect value of the compromise effect will be affected by the reference point of the reference group norm. If the preference order of the group norm is *A*≺*B*≺*C*, the group norm pressure is very small and the effective value of the compromise effect will not be significantly affected when the group norm takes product A (competitive product) as the reference point. When group norms take product B (target product) as the reference point, under the joint action of group norms and compromise effect, the effect value of compromise effect reaches the maximum, indicating that group norms will also induce compromise effect. When the group norm takes product C (temptation product) as the reference point, the probability of selecting competitive product A and target product B decreases significantly. At this time, there is a polarization phenomenon of preference for product C. Compared with compromise product B, competitive product A is farther away from the reference point of group norms and its market share decreases more, so the compromise effect still exists. According to previous research results, the change of consumer choice can be explained as the result of “coding” according to product attributes, gains, or losses of group relations under different reference points (Kahneman and Tversky, 1979; Kahneman et al., 1991). Consumers evaluate the expected results to minimize negative results through coding (Lim and Hahn, 2020).

### Theoretical Implications

Huber et al. (1982) believed that group norms may be one of the causes of context effect. Simonson (1989) was aware of the impact of reference group norms on the compromise effect, but the hypothesis has not been fully confirmed. We absorbed the research results of Bearden and Etzel (1982) on the impact of reference group norms, fully considered the two characteristics of “public” and “luxury” in the selection of experimental products, and confirmed that reference group norms will not only affect the compromise effect but also induce the compromise effect. This shows that the occurrence mechanism of the compromise effect is very complex. It can be induced not only by some factors, such as individual psychological characteristics (Simonson, 1989), material phenomenon (Hamilton et al., 2007), physiological response (Hedgcock and Rao, 2009), and consumer information processing mode (Hamilton et al., 2007) but also by social factors, such as friendship, sense of security, sense of belonging, and being respected by others.

Simonson (1989) believes that the mechanism of compromise effect may be due to the complexity and difficulties faced by consumers in the decision-making process, which affects the preference fluency of consumers. Novemsky et al. (2007) also believe that preference fluency leads to a compromise effect by affecting consumers’ attitude toward risk and the trade-off between “price and quality,” or by affecting the objective conditions of the selection set (such as cognitive load and variety). By increasing the complexity and difficulty of consumer decision-making, that is, expanding consumer decision-making from a single product reference point to two reference points of “product attribute + social impact,” we find that consumers always follow the principle of prevention-focused (Pham and Higgins, 2005), and take avoiding wrong selection as the core starting point of choice, that is, consumers are always loss averse. Therefore, we believe that the generation mechanism of consumer compromise effect is more due to the correction of consumer’s purchase motivation. For example, when the group norms take restaurants B and C as the reference points, respectively, the market share of restaurant A decreases significantly, which indicates that consumers have gradually transferred their purchase motivation to the group norms. This shows that the occurrence or change of the compromise effect is more likely to be the result of the correction of consumers’ purchase motivation. In this sense, the conclusion of this article supports that the compromise effect is induced by motivation (Malaviya and Sivakumar, 2002; Mourali et al., 2007).

Another question worth discussing is whether the standardization of reference groups would weaken the rationality of consumers’ purchase behavior. We found that there is a polarization of choice when taking restaurant C (luxury goods) as the reference point. From the perspective of product attributes, group standardization does weaken the rationality of consumers’ purchase behavior (Rook and Fisher, 1995). However, from the perspective of social relationship value acquisition, it is rational. Consumers make decisions in accordance with group norms and gain more face benefits. It is shown that what consumers need is not the product itself, but the problems that the product can solve and the social significance given by the product.

### Practical Implications

First, if the company takes the compromise effect as the competitive strategy of its products, it is necessary to construct an effective product perception map around the target group. The company must clarify which products are competitive products and which products can be used as temptation products, to build a product set that can produce the psychological perception of compromise effect, and take it as the direct basis for the company’s product strategy formulation, advertising delivery, product display, and price promotion.

Second, the market often understands the norm impact as high price and high quality. If the compromise effect is established, the use of a “price-quality heuristic” for the target product can effectively improve the effect of the compromise effect. According to the research of Hoch and Deighton (1989), when consumers can only judge the quality (if the quality is related to identity consumption and face consumption) based on price, the effect of norm influence should be better. “Love her, treat her Haagen Dazs,” an advertisement of Haagen Dazs in China, has adopted a high-price strategy with normative advertising and achieved good results.

Third, the impact of norms is unstable, and consumers are unlikely to internalize forced behavior. Enterprises should pay special attention to the limitations of using group norms to affect the compromise effect. The most important premise of using norms is to internalize group values and norms (Bearden and Etzel, 1982; Ito et al., 1998), that is, norms should transform consumer’s purchase of products from external motivation to internal motivation. This requires that the products sold by enterprises to consumers are not only the products themselves but also give specific social significance to the purchase behavior according to the product use scenario, to trigger the emotion of consumers.

### Limitations and Future Research

Following the general research paradigm of compromise effect, this study deliberately obscures the real name of restaurants to make the research simpler, but this may also lead to research bias. First, an important clue of group norms affecting consumers is the brand name. Second, to avoid expected regret, consumers often make decisions based on product popularity when choosing products in product concentration (Simonson and Tversky, 1992). In addition, this study only analyzes the norm impact of identity reference groups and does not consider the dissociative reference groups.

## Data Availability Statement

The raw data supporting the conclusions of this article will be made available by the authors, without undue reservation.

## Ethics Statement

Ethical review and approval was not required for the study of human participants in accordance with the local legislation and institutional requirements. The patients/participants provided their written informed consent to participate in this study.

## Author Contributions

QD contributed to designing, analyzing, and writing the study. KL contributed to collecting research data and writing the study. Both authors contributed to the article and approved the submitted version.

## Conflict of Interest

The authors declare that the research was conducted in the absence of any commercial or financial relationships that could be construed as a potential conflict of interest.

## Publisher’s Note

All claims expressed in this article are solely those of the authors and do not necessarily represent those of their affiliated organizations, or those of the publisher, the editors and the reviewers. Any product that may be evaluated in this article, or claim that may be made by its manufacturer, is not guaranteed or endorsed by the publisher.
